# Supplementary material for: Predicting CRISPR-Cas12a guide efficiency for targeting using machine learning
Source: PLoS One. 2023 Oct 17;18(10):e0292924. doi: 10.1371/journal.pone.0292924 (PMC10581463; doi:10.1371/journal.pone.0292924)
Supplement: S1 Fig — The ratio of Cas9 to Cas12a predicted off-targets across the genomes for each gRNA designed for (a) low GC content (30.8%) and (b) high GC content (66%) with zero to five mismatches to the original target and aggregated per genome. A ratio > 1 signifies Cas9 off-targets are higher than Cas12a. Statistical tests were performed using a non-parametric test (Kruskal-Wallis) and multiple comparisons were corrected with a Bonferroni test. ** indicates corrected p < 0.01 and *** indicates corrected p < 0.001 n = 10 (low GC content) and n = 18 (high GC content) per group. (PDF) [file pone.0292924.s001.pdf]

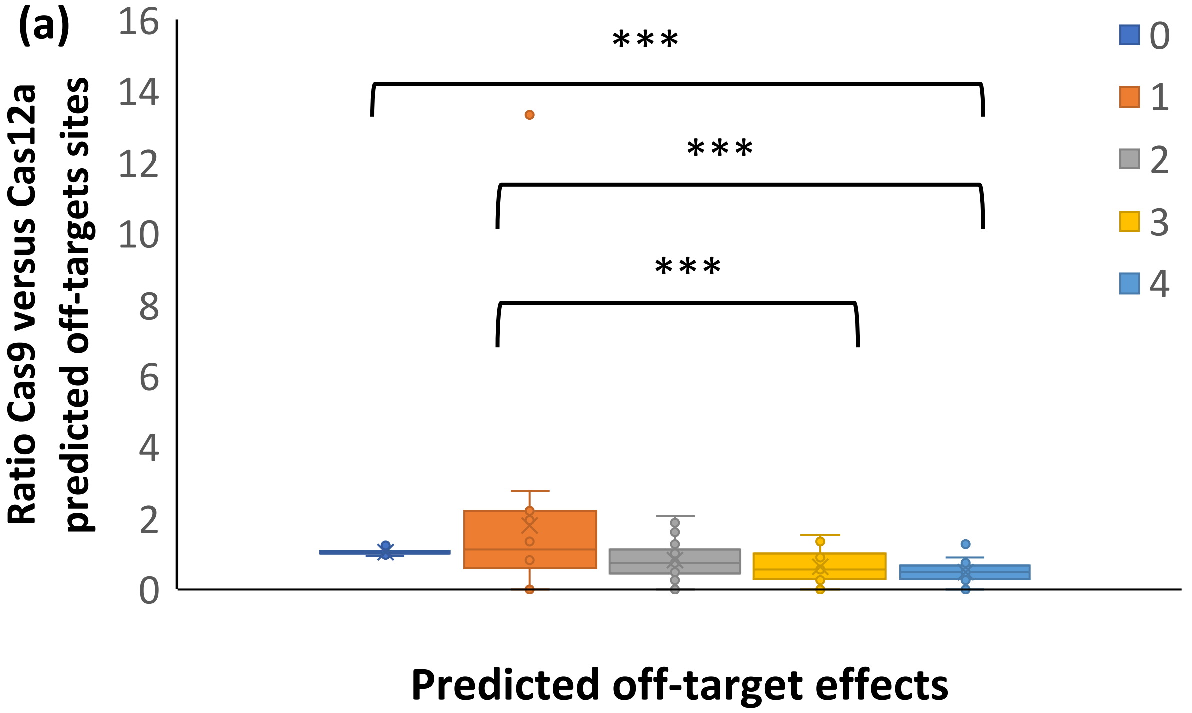

(a)
16
14
12
10
8
6
4
2
0
Ratio Cas9 versus Cas12a predicted off-targets sites
***
***
***
0
1
2
3
4
Predicted off-target effects

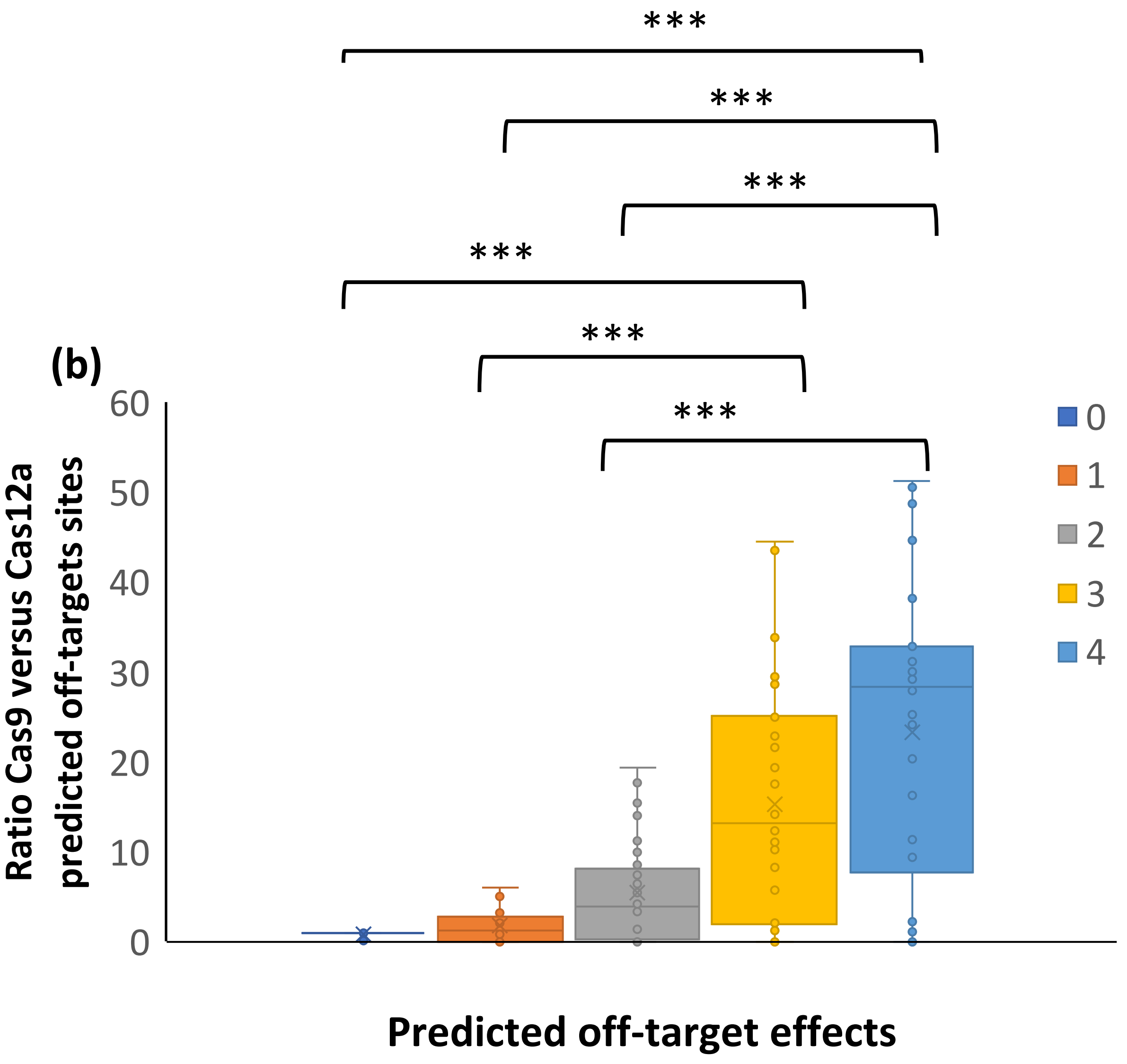

***
***
***
***
***
***
(b)
60
50
40
30
20
10
0
Ratio Cas9 versus Cas12a predicted off-targets sites
0
1
2
3
4
Predicted off-target effects

Suppl fig 1
